# Eutectogel-Based Drug Delivery: An Innovative Approach for Atenolol Administration

**DOI:** 10.3390/pharmaceutics16121552

**Published:** 2024-12-04

**Authors:** Roberta Cassano, Roberta Sole, Carlo Siciliano, Noemi Baldino, Olga Mileti, Debora Procopio, Federica Curcio, Gabriella Calviello, Simona Serini, Sonia Trombino, Maria Luisa Di Gioia

**Affiliations:** 1 Dipartimento di Farmacia, Salute e Scienze della Nutrizione, Università della Calabria, Arcavacata di Rende, 87036 Cosenza, Italy; roberta.cassano@unical.it (R.C.); roberta.sole@unical.it (R.S.); carlo.siciliano@unical.it (C.S.); debora.procopio@unical.it (D.P.); federica.curcio@unical.it (F.C.); 2 Dipartimento di Ingegneria Informatica, Modellistica, Elettronica e Sistemistica, Università della Calabria, Arcavacata di Rende, 87036 Cosenza, Italy; noemi.baldino@unical.it (N.B.); o.mileti@dimes.unical.it (O.M.); 3 Dipartimento di Medicina e Chirurgia Traslazionale, Sezione di Patologia Generale, Facoltà di Medicina e Chirurgia, Università Cattolica del Sacro Cuore, Largo F. Vito, 00168 Roma, Italy; gabriella.calviello@unicatt.it (G.C.); simona.serini@unicatt.it (S.S.); 4 Fondazione Policlinico Universitario A. Gemelli IRCCS, Largo F. Vito, 00168 Roma, Italy

**Keywords:** deep eutectic solvents, green solvents, atenolol, eutectogel, drug delivery, β-blockers, antihypertensive drugs

## Abstract

**Background:** Hypertension affects 32% of adults worldwide, leading to a significant global consumption of cardiovascular medications. Atenolol, a β-adrenergic receptor blocker, is widely prescribed for cardiovascular diseases such as hypertension, angina pectoris, and myocardial infarction. According to the Biopharmaceutics Classification System (BCS), atenolol belongs to Class III, characterized by high solubility but low permeability. Currently, atenolol is commercially available in oral formulations. Increasing attention is being directed towards developing cost-effective transdermal delivery systems, due to their ease of use and better patient compliance. Eutectogels represent next-generation systems that are attracting great interest in the scientific community. Typically obtained from deep eutectic solvents (DESs) combined with gelling agents, these systems exhibit unique properties due to the intrinsic characteristics of DESs. **Methods:** In this study, a DES based on choline chloride as a hydrogen bond acceptor (HBA) and propylene glycol as a hydrogen bond donor (HBD) was explored to enhance the topical delivery of atenolol. The solubility of atenolol in the DES was evaluated using spectroscopic and thermodynamic measurements which confirmed the formation of hydrogen bonds between the drug and DES components. Additionally, the safety of the DES was assessed in a cell viability assay. Subsequently, we formulated eutectogels with different concentrations using animal gelatin and Tego Carbomer 140, and characterized these formulations through rheological measurements, swelling percentage, and permeation studies with Franz cells. **Results:** These novel eutectogels exhibit superior performance over conventional hydrogels, with a release rate of approximately 86% and 51% for Carbomer- and gelatin-based eutectogels, respectively. In contrast, comparable hydrogels released only about 27% and 35%. **Conclusions:** These findings underscore the promising potential of eutectogels for the transdermal delivery of atenolol.

## 1. Introduction

Hypertension, affecting approximately 32% of the adult population aged 30–79 years, is one of the most widespread diseases in industrialized countries and constitutes one of the major clinical problems [1,2,3]. It is also referred to as the “silent killer” due to its asymptomatic nature and potential for serious complications. Prolonged, untreated hypertension can lead to atherosclerosis, heart attack, stroke, aneurysms, heart failure, hypertensive nephropathy, hypertensive retinopathy, and cognitive deficits [4]. Atenolol (ATL), chemically (RS)-4-(2-hydroxy-3-isopropylaminopropoxy)phenylacetamide, a β-adrenergic receptor blocker, is commonly prescribed for hypertension and heart disease [5,6,7]. Atenolol works by selectively binding to β1-adrenergic receptors present in vascular smooth muscle and the heart, blocking the inotropic and positive chronotropes of endogenous catecholamines, thus inhibiting sympathetic stimulation. This activity causes a reduction in heart rate and blood pressure, and decreases myocardial contractility [7].

Although typically administered orally in dosages ranging from 25 mg to 100 mg [8], the oral administration of atenolol can cause adverse effects such as diarrhea, nausea, ischemic colitis, and mesenteric arterial thrombosis. Furthermore, reduction in drug concentration on the receptor side and fluctuation in plasma drug levels have been reported [8,9,10,11]. The intravenous administration of atenolol achieves nearly 100% bioavailability and maintains stable plasma concentrations, but it requires healthcare supervision and limits patient mobility due to infection risks and needle access. For drugs with a narrow therapeutic window, in fact, continuous intravenous infusion minimizes fluctuations outside the therapeutic window, thus maintaining plasma concentrations stable. However, this route of administration requires the intervention of a healthcare worker and prolonged access to the needle, which exposes the patient to infections and limits mobility [12]. Therefore, the development of an adequate drug delivery system for antihypertensive medications capable of maintaining a consistent blood level over a prolonged period without adverse effects associated with frequent oral administration is of the outmost importance. Such a system should also ensure high patient compliance and should take into consideration the characteristics of solubility and permeability of atenolol. According to the Biopharmaceutics Classification System (BCS) [13], ATL belongs to class III, indicating high solubility but low permeability. A strategy for enhancing its permeability is essential for creating alternative formulations to oral administration. An innovative gel formulation for the transdermal delivery of atenolol, aligned with green chemistry principles, seems to be a challenge that could revolutionize hypertension treatment [14].

Deep eutectic solvents (DESs) have emerged as green solvents in recent years. They are eutectic systems prepared by mixing two or more components in a specific ratio, resulting in a depression of the melting point compared to that of the individual components [15,16,17]. DESs are attractive for a variety of applications, particularly in the chemical and pharmaceutical fields, as they offer several advantageous properties, such as biodegradability, low toxicity, versatility, ease of preparation, and compatibility with a wide range of organic and inorganic compounds [18,19,20,21]. More recently, DESs have been successfully used to prepare a new class of gels, known as eutectogels, which are complex systems composed of cross-linked polymer networks and deep eutectic solvents [22,23,24].

Various studies have been carried out to explore the possibility to employ DESs as permeation enhancers. These solvents can break the external layer of the skin responsible for its impermeability, thus allowing the non-invasive delivery of bioactive molecules via transdermal permeation [25,26,27]. In eutectogels, the permeation-enhancing properties of DESs are combined with those of gelling polymers to create a particularly stable and ductile system for drug delivery [28].

**Figure 1:**
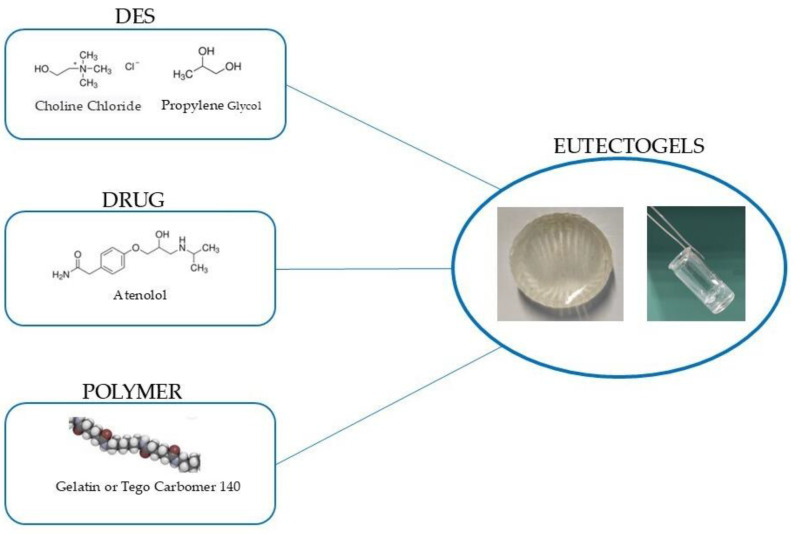
Eutectogels prepared in this study.

In this study, we propose the preparation of a DES based on choline chloride and propylene glycol to favor the solubility of atenolol. Then, we investigate the preparation of eutectogels derived from the aforementioned deep eutectic solvents, with the objective of improving the transdermal delivery of the drug. By optimizing the formulation of these eutectogels, we seek to enhance drug permeation and potential therapeutic efficacy (Figure 1).

## 2. Materials and Methods

### 2.1. Materials

Atenolol (≥98% (TLC), powder, ref A7655, Sigma-Aldrich, (St. Louis, MO, USA), choline chloride (ChCl) (98% purity, ref C1879, Sigma-Aldrich), propylene glycol, and phosphate-buffer solution (PBS) were used as received from Sigma-Aldrich (Merck kGaA, Darmstadt, Germany), without any further purification. Tego Carbomer 140 and food-grade animal gelatin 280 bloom were purchased from ACEF S.p.A. (Fiorenzuola d’Arda, PC, Italy).

### 2.2. Preparation of DES

Before preparing the DES used in this study, choline chloride was dried during 72 h to remove water absorbed from ambient humidity. Propylene glycol was placed under molecular sieves for 72 h, also to decrease its water content. Choline chloride (ChCl) and propylene glycol (PG) were used for the preparation of the DES according to Silva et al. [29]. Briefly, in a round bottom flask, ChCl and PG were mixed in a molar ratio of 1:3. The mixture was kept under constant stirring at 60 °C, until a clear liquid was formed.

### 2.3. Polarized Optical Microscopy (POM) Analysis

DES samples containing progressive amounts of ATL were placed onto a microscope slide for examination under 10× magnification. The polarized light image was observed at room temperature with a Nikon ECLIPSE LV100N polarizing microscope (Nikon Corporation, Tokyo, Japan) coupled with a Nikon DS-Fi2 camera. When a solid crystalline structure is absent, the polarized light image appears uniformly black [30].

### 2.4. DES Solubility Measurement

To evaluate the solubility of ATL in the DES, an excess amount of ATL was introduced into ~1 mL of the eutectic mixture. Subsequently, the mixture was continuously stirred for 24 h, to achieve equilibrium conditions at room temperature. The saturated sample was filtered using a PTFE syringe filter with a 0.45 μm membrane, to separate the macroscopic solid from the liquid phase. Then, the liquid phase was diluted with ethanol, and the amount of the dissolved drug was determined using a validated UV method on a UV spectrophotometer (UV-530, JASCO Corporation, Hachioji, Tokyo, Japan). The absorbance of the solutions was measured at the API maximum absorption wavelength (273 nm). The concentration of ATL was assessed on a calibration curve obtained by dissolving pre-weighed amounts of the drug in ethanol and measuring their absorbance as a function of concentration, expressed as the mass of the drug dissolved in mL of the solvent (mg/mL).

### 2.5. Fourier-Transform Infrared Spectroscopy (FTIR) Analysis

The DES, the pure drug, and the drug dissolved in the DES were analyzed by FTIR spectroscopy (Perkin-Elmer 1720 FTIR, Perkin Elmer company, Waltham, MA, USA) at a resolution of 4 cm^−1^ in the wavelength range of 4000 to 400 cm^−1^.

### 2.6. Differential Scanning Calorimetry (DSC) Analysis

DSC experiments were performed for the different samples: the DES, the drug and the DES with the drug. Briefly, 5–9 mg of the different samples was placed in hermetically closed sample pans before thermal analysis using the DSC instrument (DSC 200 PC, Netzsch-Gerätebau GmbH, Selb, Germany). The thermograms were collected from 20 to 300 °C at a rate of 5 °C min^−1^, to detect all the transitions, melting points, and T_g_ of the substances.

### 2.7. NMR Analysis

NMR measurements were performed on a Bruker Advance 300 Ultra shielded spectrometer, operating at 300.132 and 75.08 MHz for ^1^H and ^13^C, respectively. The instrument was equipped with a 5 mm BB0 probe with *Z*-axis gradient coils and an automatic temperature control unit. NMR 5 mm tubes (Wilmad-LabGlass Industries, Vineland, NJ, USA) were used for all samples. Spectra were obtained by setting the temperature at 25 °C, with a variation of ±0.1 °C. Chemical shifts were expressed in ppm and referred to the signal of dimethyl sulfoxide (central line of the quintet at 2.51 ppm for proton spectra, and central line of the septet at 40.00 ppm for carbon analysis). All spectra were obtained by applying pulse sequences from Bruker pulse program libraries. Acquisition and elaboration parameters were as elsewhere published [31].

Choline Chloride (ChCl). ^1^H NMR (δ): 5.70 (t, *J* = 6 Hz, 1H, OH), 3.75–3.87 (m, 2H, CH_2_O), 3.35–3.47 (m, 2H, CH_2_N^+^), 3.17 (s, 9H, CH_3_) ppm; ^13^C NMR (δ): 67.33, 96.42, 53.51 ppm.

Atenolol (ATL). ^1^H NMR (DMSO-*d*_6_), δ: 7.39 (bs, 1H; NH_2_), 7.16 (d, *J* = 6 Hz, 2H; ArH), 6.86 (d, *J* = 6 Hz, 3H; ArH and NH_2_), 4.97 (bs, 1H, OH), 3.76–3.89 (2 m, 3H; CHOH and OCH_2_), 3.29 (s, 2H, CH_2_CO), 2.63–2.76 (m, 2H; CH_2_NH and NHCH), 2.49–2.61 (m, 1H; CH_2_NH), 1.50 (bs, 1H; NH), 0.98 (d, *J* = 6 Hz, 9H; CH_3_) ppm; ^13^C NMR (δ): 173.6 (CO), 157.9 (CAr), 130.6 (CHAr), 128.9 (CAr), 114.8 (CHAr), 79.9 (CH_2_O), 71.3 (CHOH), 50.7 [CH(CH_3_)], 48.8 (CH_2_NH), 42.1 (CH_2_CO), 23.4 (CH_3_) ppm. FT-IR: 1237.49; 1417.41, 1515.55; 1634.49, 2964.81, 3199.76, 3349.76 cm^−1^

(ChCl:PG) DES. ^1^H NMR (δ): 5.55 (t, *J* = 3 Hz, 1H, OH ChCl), 4.57 (t, *J* = 6 Hz, 1H, CH_2_OH PG), 4.51 (d, *J* = 3 Hz, 1H, CHOH PG), 3.75–3.87 (m, 2H, CH_2_OH ChCl), 3.48–3.62 (m, 1H, CH PG), 3.40–3.47 (m, 2H, CH_2_N^+^ PG), 3.20–3.32 (m, 1H, CH_2_ PG), 3.15 (s, 9H, CH_3_ ChCl), 3.10–3.20 (m, 1H, CH_2_ PG), 0.99 (d, *J* = 6 Hz, 3H, CH_3_ PG) ppm; ^13^C NMR (δ): 67.78, 67.44, 55.60, 53.64, 20.48 ppm.

(ChCl:PG) DES-ATL. ^1^H NMR (δ): 7.65 (br s, 1H, NH_2_CO ATL), 7.13 (d, 2H, *J* = 9 Hz, ArH ATL), 6.89 (br s, 1H, NH_2_CO ATL), 6.81 (d, 2H, *J* = 9Hz, ArH ATL), 5.42 (br s, OH ChCl), 4.65, (br s, 2H, OH PG, OH PG), 3.80 (m, 4H, CH_2_ ChCl and CH_2_OH PG), 3.57–3.55 (m, 4H, CHOH and OCH_2_ ATL, CHOH PG), 2.52–2.49 (m, 3H, CH_2_NH, NHCH), 3.44 (m, 2H, CH_2_ ChCl), 3.23 (m, 2H, CH_2_CO, (s, 3.31 (s, 9H, N(CH_3_)_3_, 0.96 (d, *J* = 7.20 Hz, CH(CH_3_)_2_) ppm; ^13^C NMR (δ): 174.27, 158.08, 130.89, 115.08, 69.51, 68.95, 68.67, 68.59, 68.95. 68.28, 67.96, 56.00, 54.06, 52.85, 20.62, 19.76 ppm.

### 2.8. In Vitro Studies: Cell Line and Treatments

The human monocytic cell line THP-1 was obtained from AddexBio Technologies (San Diego, CA, USA). The cells were maintained in RPMI culture medium containing glutamine (2 mM), HEPES (10 mM), sodium pyruvate (1 mM), glucose (4.5 g/L), sodium bicarbonate (1.5 g/L), β-mercaptoethanol (0.05 mM), and fetal bovine serum (FBS, 10%). The immortalized human keratinocytes NCTC 2544 were kindly donated by Dr. R. De Bellis (University of Urbino, Italy) and cultured in DMEM culture medium containing glutamine (2 mM), antibiotics (Penicillin, 100 U/mL, and Streptomycin, 100 µg/mL), and FBS (10%). Both cell types were maintained at 37 °C in a humidified atmosphere with 5% CO_2_. The cells were kept in an exponential growth phase by seeding them twice a week at a concentration of 3 × 10^5^ cells/mL. The DES-based lyophilized samples were solubilized in the respective culture media of NCTC 2544 and THP-1 cells. Aliquots of the stock solutions were taken to achieve final culture concentrations of 0.5, 1, and 5 µg/mL.

#### Cell Viability Assay Using MTT Assay In Vitro

The MTT assay was used to assess cellular metabolic activity as an indicator of cell viability and verify if the DES was safe and non-toxic. This colorimetric method is based on the reduction of the tetrazolium salt 3-(4,5-dimethylthiazol-2-yl)-2,5-diphenyltetrazolium bromide (MTT), a yellow compound, to blue formazan crystals by metabolically active cells. Viable cells contain the mitochondrial enzyme succinate dehydrogenase, which converts the MTT tetrazolium ring (yellow) to formazan (blue). The insoluble formazan crystals are dissolved using dimethyl sulfoxide (DMSO), and the resulting colored solution is quantified by measuring absorbance at 570 nm using a plate spectrophotometer. The greater the color intensity of the solution, the higher the number of metabolically active viable cells. NCTC 2544 and THP-1 cells were seeded at a concentration of 5 × 10^3^ cells/well in 96-well multiwell plates in a final volume of 200 µL/well. After 24 h, the culture medium was removed and replaced with fresh medium containing the DES compound or control. At increasing time intervals (24–72 h), 50 µL of MTT solution (2 mg/mL in PBS) was added to each well, and the culture plates were further incubated at 37 °C for four hours. Subsequently, the supernatant was removed, and the formazan crystals formed were solubilized using DMSO (100 µL/well). Absorbance was measured at 570 nm and 630 nanometers (as baseline absorbance to be subtracted from that measured at 570 nm) using a plate spectrophotometer (Tecan, Männedorf, Switzerland). Cell viability was calculated using the following formula:

(1) $$\%\;cells\;viability=\frac{Absorbance\;of\;treated\;cells}{Absorbance\;of\;control\;cells}\times 100$$

### 2.9. Preparation of Eutectogels

Various natural gelling agents were evaluated for the preparation of eutectogels, based on a previous work by Abbott et al. [32]. Tego Carbomer 140 and animal gelatin were the most effective among the tested gelling agents. The two types of gels were prepared using two different methods. For the gel composed by DES and Tego Carbomer 140, different concentrations (2%, 3%, and 4% *w*/*w*) were tested. Tego Carbomer 140 was added to the DES–drug solution (containing 12 mg of drug) under constant stirring at room temperature until gel formation was complete. In the DES–gelatin formulation, gelatin was added to the DES at 60 °C under constant stirring. Once a homogeneous phase was achieved, the heat was turned off, and 12 mg of the drug was incorporated. The mixture was then poured into a mold and refrigerated overnight to allow gel formation. Three different concentrations (10%, 15%, and 20% *w*/*w*) were prepared. These formulations were compared with control gels, where the drug was solubilized in water instead of DES, following the same preparation method.

### 2.10. Rheological Study

The eutectogels were characterized by rheological tests, using a rotational rheometer (HAAKE MARS III, Thermo Fisher Scientific, Bremen, Germany) equipped with a parallel plate geometry (ϕ = 20 mm, gap = 1.3 ± 0.1 mm) and a Peltier system to control the temperature. Silicone oil (20 cSt, VWR Chemicals, Fontenai-sous-Bois, France) was used to avoid water evaporation phenomena during tests. Frequency sweep tests at 25 °C and 37 °C, in the linear region, were performed to investigate the material microstructure. The LVR (linear viscoelastic region) was individuated by the stress sweep tests performed at 25 and 37 °C. A temperature ramp test (time cure) was performed in linearity from 20 to 40 °C for the gelatin samples and from 20 to 60 °C for the carbomer samples, using a heating rate of 2 °C/min, to study the thermal stability of the samples. The resulting data were interpreted in terms of complex dynamic modulus, G*, and phase angle, δ [33].

The frequency sweep tests were interpreted using the weak gel model, a model widely used for samples weakly structured as gels, emulsions, doughs, and also interfacial gels [33,34,35]:

(2) $$G^{*}=A\omega^{\left(\frac{1}{Z}\right)}$$

where A is a measure of gel strength and z of the gel structuration.

### 2.11. Water Regain Study

This study is used to assess the affinity of the eutectogels towards aqueous media and thus determine the swelling percentage (WR%) until swelling equilibrium is reached. Each gel sample was weighed (50 mg) and placed inside a glass filter with a porosity of 2 μm, the latter previously wetted with a buffer solution (PBS 0.001 M, pH 7.4) simulating the conditions of biological fluids, centrifuged at 3000 rpm for 5 min, and finally weighed. Afterwards, the gel and the filter were weighed again and then immersed in phosphate buffer.

At predefined time intervals (1 h, 2 h, 3 h, 4 h, 6 h, 8 h, and 24 h), the filters containing the gels were weighed to monitor the weight variation from the initial value, i.e., that corresponding to the dry gel [36].

To this end, the excess PBS was removed by centrifuging the filters at 3000 rpm for 5 min, which were weighed, and the percentage of swelling was determined using the following formula:

(3) $$WR\%=\frac{Ws-Wd}{Wd}\times 100$$

where WR% is the percent of water regain; Ws is the weight of the swollen gel; Wd is the weight of the dried gel.

The experiments were conducted in triplicate.

### 2.12. Permeation Study

A permeation study was performed in triplicate (n = 3) using cellulose acetate membranes and Franz diffusion cells. The membrane was allowed to equilibrate with a dissolution medium (pH 7.4 buffer solution) for 1 h prior to sample application. The receptor compartment was filled with 8 mL of the pH 7.4 buffer solution.

The gel sample was placed in the donor compartment, on top of the membrane, and covered with laboratory film (Parafilm^®^). The entire system was maintained at a temperature of 37 ± 0.5 °C to mimic physiological conditions. At specific time intervals (1, 2, 3, 4, 6, 24, 48, and 72 h), 8 mL of receiver solution was withdrawn and replaced with a fresh buffer. The concentration of atenolol in the receiver solution samples was assayed using UV–Vis spectrophotometry [37].

### 2.13. Statistical Analysis

The data obtained were analyzed using one-way analysis of variance (ANOVA) followed by Tukey’s test (InStat GraphPad software 10.4.0.).

## 3. Results and Discussion

### 3.1. Preparation of the DES

Several methods have been proposed for the preparation of DESs, such as vacuum drying, heating and stirring, and freeze-drying [38]. Heating and stirring was the method employed in this study, due to its simplicity and widespread usage. Choline chloride was chosen as the hydrogen bond acceptor, and propylene glycol as the hydrogen bond donor. Choline chloride is a complex B vitamin that plays a vital role in cellular metabolism, commonly used as a food additive [39]. Propylene glycol is a viscous liquid recognized in the pharmacopeia as an excipient for the formulation of dermatological preparations. Additionally, it is used as a non-toxic polyol in food processing and polymer production [40]. The chosen components are non-toxic and make the DES advantageous for its applications as drug delivery systems, given its natural and therapeutic properties as well as biocompatibility. In this study, we selected a 1:3 molar ratio of ChCl to PG based on the existing literature which suggests that this molar ratio facilitates the formation of a stable liquid DES at room temperature. The stability and liquidity at ambient conditions are essential criteria for DES applications, allowing for ease of handling and broader potential uses [41]. To confirm the formation of eutectic mixtures, POM and DSC measurements were conducted. The prepared DESs were characterized by DSC, FT-IR, and NMR analysis (see Supplementary Materials). The results were consistent with the literature [30,42].

### 3.2. DES Solubility Assay

DESs have been reported as a promising alternative to improve the solubility and/or permeability of drugs and modulate their bioavailability [43]. Therefore, in this study, the potential of this DES in improving the solubility of ATL was explored. The shake flask method represents a standard method for determining the solubility of drugs in a solvent [44]. An excess amount of the drug is added to a given volume of solvent, and the mixture is subjected to constant stirring at room temperature until equilibrium is reached. Once equilibrium is achieved, the undissolved solid is separated from the solution, and the concentration of the dissolved drug is measured by an appropriate method. Our results showed that the solubility of ATL was approximately 15 mg/mL. To detect intermolecular interactions between ATL and the DES components, DSC, FIT-IR, and NMR analyses were performed for the pure atenolol drug, the individual DES components, and the drug–DES mixtures. The successful dissolution of ATL in the DES system was confirmed by POM images, as no crystal-like structures were observed. The solubility of the drug in the DES represents the maximum quantity that can be loaded into the matrix.

### 3.3. DSC Analysis

**Figure 2:**
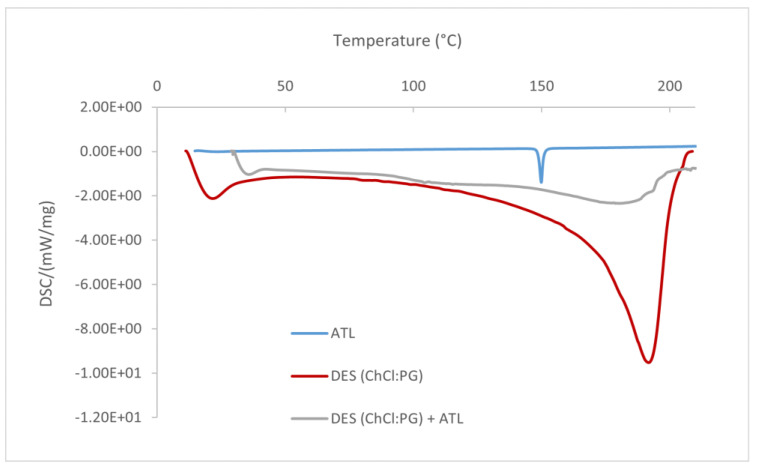
DSC thermograms of atenolol (ATL), ChCl:PG DES, and drug–DES mixture.

Figure 2 reports the DSC analysis of pure ATL, ChCl:PG DES, and the ATL–DES mixture. The thermogram of the ChCl:PG DES represents a complex formed by hydrogen bonding between the starting components.

The DSC thermogram of pure ATL (blue line) shows a clear endothermic melting peak at 149.86 °C, indicating the extremely crystalline nature of the drug. In the case of the eutectic mixture with the drug (gray line), the clear peak is absent, indicating that the ATL is present in an amorphous state stabilized by the weak interactions that are established between the drug and DES. The loss of the ordered structure also occurs in the DES [45,46]. This is confirmed by the comparison of the thermogram of pure DES (red line) which is characterized by a pronounced peak at the temperature of 191.89 °C which in the mixture is less evident and is broader. Consequently, it is possible to affirm the successful solubilization of atenolol in the DES.

### 3.4. FT-IR Analysis

To acquire a comprehensive understanding of the intermolecular interactions between ATL and the components of the ChCl:PG DES, the infrared spectra of pure ATL, the DES, and the drug–DES system were carried out.

**Figure 3:**
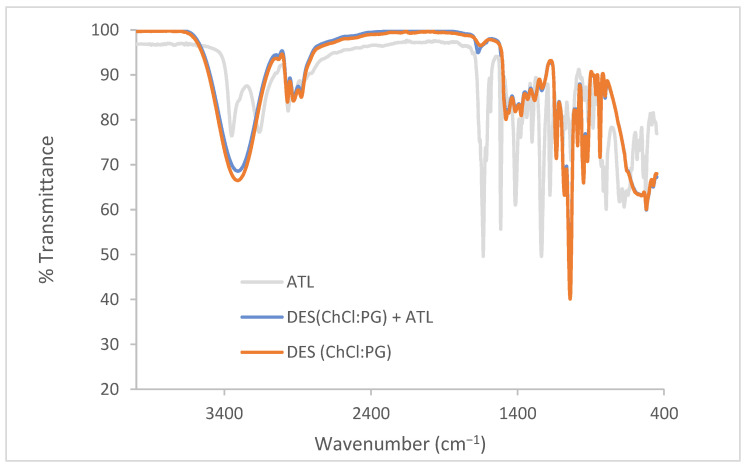
FT-IR analysis of (ChCl:PG) DES, atenolol (ATL), and (ChCl:PG) DES with atenolol.

Figure 3 shows the FT-IR spectra of the ChCl:PG DES, the pure drug ATL, and the ATL–DES system. The main differences can be observed in the 2800–3600 cm^−1^ and the 1670–1600 cm^−1^ regions. The first region displays the elongation vibrations of hydroxyl groups (-OH) of the DES components and the stretching of the amino group (N-H) of ATL. The expansion and displacement of these bands are ascribed to the establishment of hydrogen bonds among the constituents. In the second region, the characteristic peak due to the carbonyl group of the drug ATL (around 1634 cm^−1^) is present. Indeed, the pure ATL exhibits a significantly more intense peak due to the carbonyl group (1671 cm^−1^) compared to the DES combined with ATL, whereas it is absent in the pure DES. These spectra confirm that the drug retains its molecular integrity upon interaction with the components of the DES.

### 3.5. NMR Characterization

The NMR spectra recorded were obtained from samples dissolved without pretreatment in deuterated dimethyl sulfoxide (DMSO-*d*_6_) as the solvent. The choice of DMSO-*d*_6_ is due to its good solubility of the active ingredient atenolol and the components of the DES, as well as its weak interaction effect with the latter.

**Figure 4:**
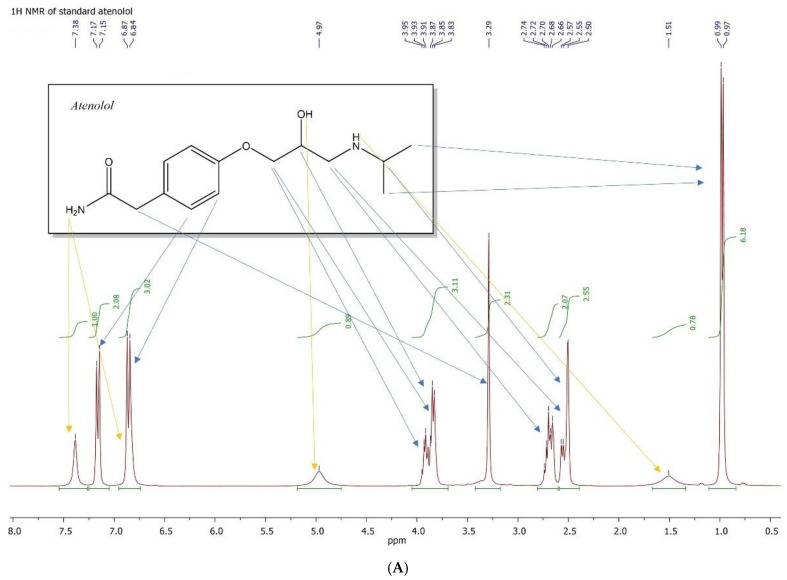

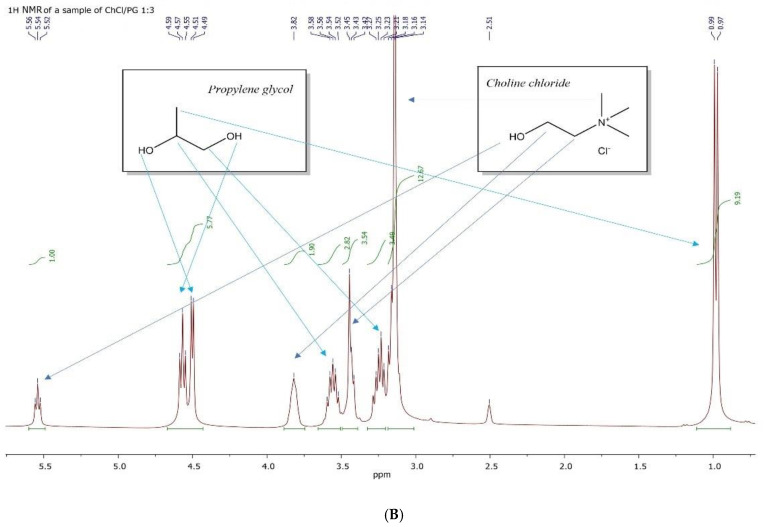
^1^H NMR spectra of atenolol (**A**) and DES (**B**).

The molecular structure of both the drug and the DES alone, as well as the drug loaded into the DES, was investigated and confirmed using proton and carbon NMR. Specifically, the proton spectrum of atenolol revealed a single visible signal at approximately 7.40 ppm in the form of a broad singlet for one of the amide protons, while the signal attributed to the second of these protons was not visible, due to overlap with the doublet at around 6.75 ppm, generated by the aromatic protons in the ortho position relative to the ether oxygen bond of the rest of the molecular structure. The proton spectrum of the DES (Figure 4) exhibited all the expected resonance signals for the ordered molecular system and highlighted relative intensities proportional to the molar composition of the binary mixture. The multiplicities of the alcoholic protons, primary and secondary, of the OH groups of propylene glycol (PG), and of the OH group of the choline molecule are noteworthy. These protons, as will be seen shortly, are assumed to be responsible for the establishment of the hydrogen bond network that stabilizes the molecular aggregate of the DES, thereby generating its particular physicochemical characteristics.

**Figure 5:**
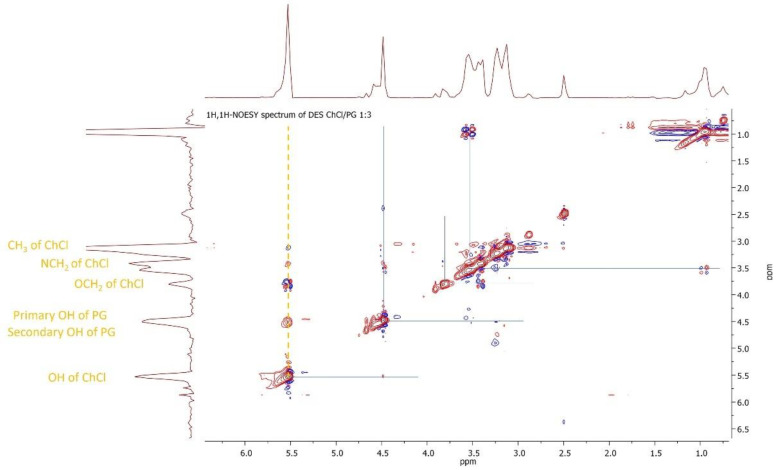
Homonuclear two-dimensional spectrum of the ChCl:PG DES.

The high-resolution homonuclear two-dimensional spectrum of the DES highlights the protons responsible for the hydrogen bond network (Figure 5). It can be noted that all the OH signals are involved in spatial correlations (yellow dashed vertical line) in the third dimension with the methylene and methyl protons of the choline molecule, thus confirming the formation of donor–acceptor pairs held together by chloride anions.

**Figure 6:**
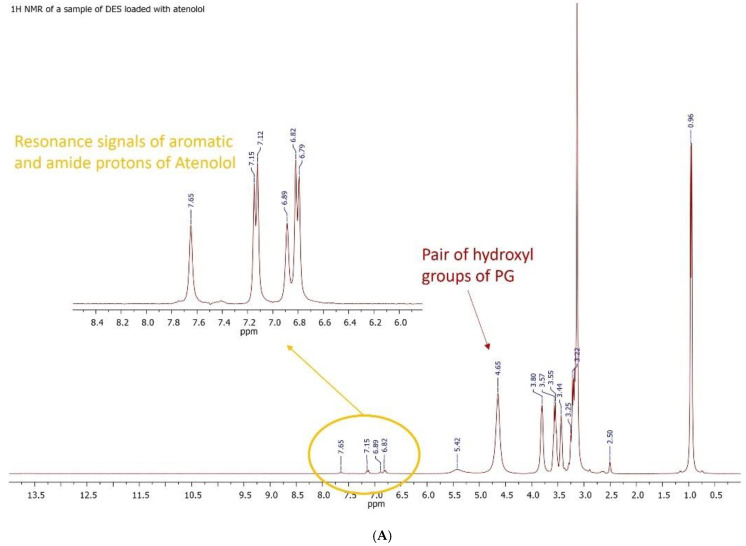

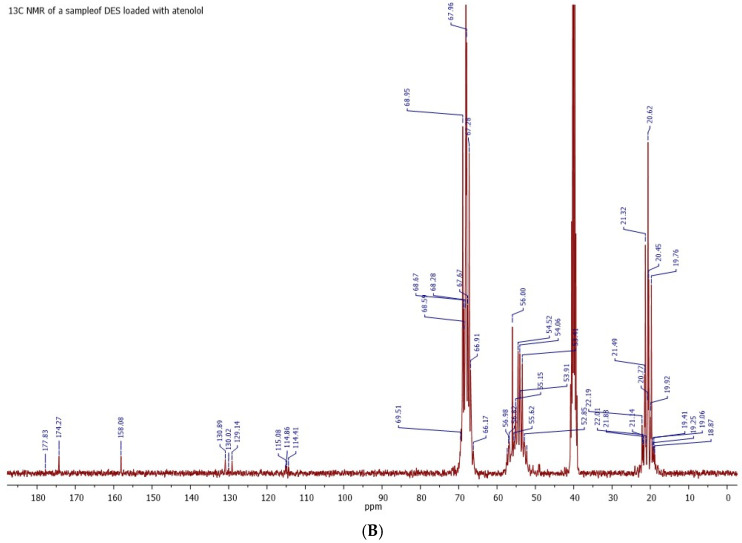
^1^H NMR spectra of (ClCh:PG)DES-ATL (**A**) and ^13^C NMR spectra of (ClCh:PG)DES-ATL (**B**).

Finally, the proton NMR spectrum recorded on a sample of the atenolol loaded-DES (Figure 6) clearly highlighted the signals of the aromatic ring spin system of the drug molecule (enlargement of the spectral window delimited by the yellow oval). Surprisingly, the two amide protons of atenolol showed two signals at 6.89 and 7.65 ppm (Figure 6A), easily distinguishable, attributable, and in a down-field position compared to what is observable in the proton spectrum of the pure drug standard (Figure 4A). This confirms the hypothesis that the amide protons of atenolol also participate in the establishment of hydrogen bonds that stabilize the ordered molecular aggregate of the DES/drug. The structural analysis of the aggregate was completed using ^13^C NMR spectroscopy (Figure 6B), which confirmed its composition, also clearly highlighting the signals attributable to the carbons of the aromatic ring of atenolol (spectral range between 110 and 180 ppm).

### 3.6. Cell Viability Assay Using MTT Assay In Vitro

**Figure 7:**
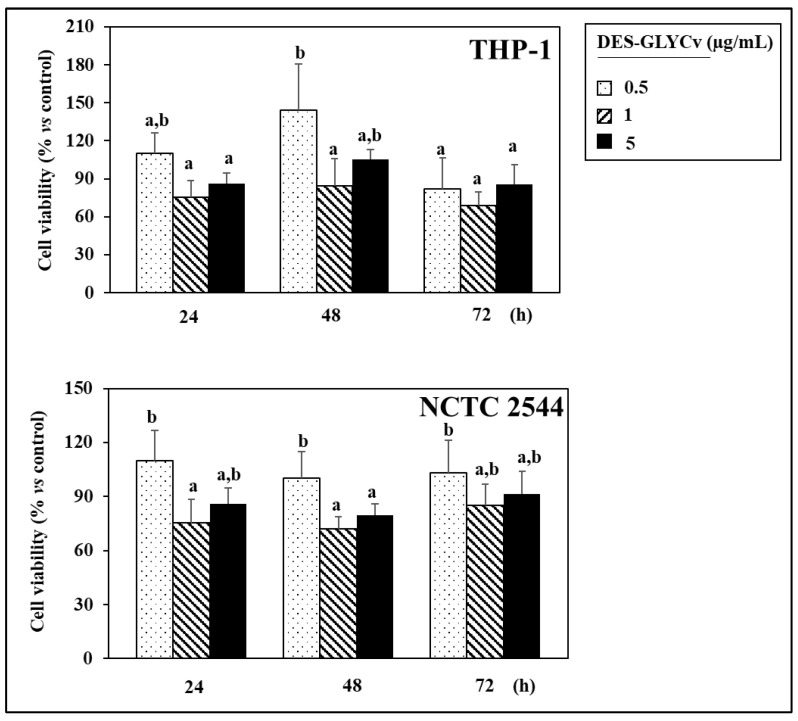
The effect of increasing concentrations (0.5–5 µg/mL) of DES on the viability of human THP-1 monocytes and immortalized human NCTC 2544 keratinocytes treated for various periods (24–72 h). Values not sharing the same lowercase letter are significantly different (*p* < 0.05, one-way ANOVA followed by Tukey’s test).

As observed in Figure 7, treatment with the DES does not exert a cytotoxic effect on THP-1 cells at any of the concentrations used. Interestingly, at the lowest concentration of DES (0.5 µg/mL), it even enhances monocyte viability (+44.5%, *p* < 0.05) after 48 h of treatment compared to control cells. However, concerning immortalized NCTC 2544 keratinocytes, we observed a significant reduction in cell viability of 9 to 28% (*p* < 0.05) in cells treated with DES at the higher concentrations used in the study (1 and 5 µg/mL) compared to control cells. This cytotoxic effect, however, was not observed when cells were treated with the lowest concentration (0.5 µg/mL). These results suggest that it would be advisable to use this DES for the delivery of active compounds at concentrations not exceeding 0.5 µg/mL to avoid any cytotoxic effects.

### 3.7. Preparation of the Eutectogels

**Figure 8:**
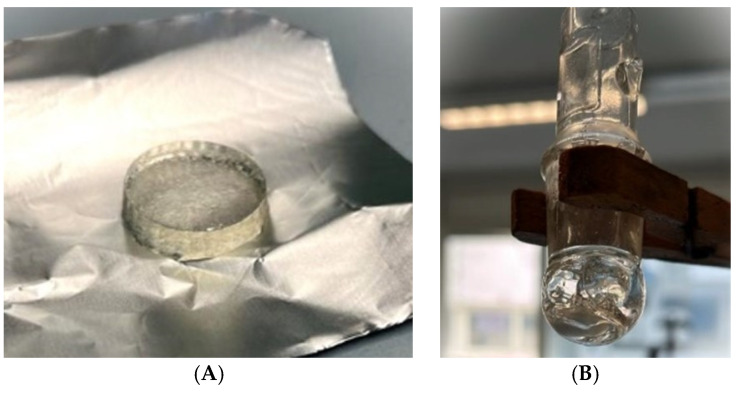
The new eutectogels prepared: (**A**) gelatin-based eutectogel; (**B**) carbomer-based eutectogel.

The proposed eutectogels were prepared following the methodology described previously [25]. Visually, as highlighted both in Figure 8A,B, their successful preparation can be confirmed. In the following paragraphs, the analyses conducted to characterize these materials will be discussed. If the maximum amount of the drug were to be carried, which, as was assessed in the previous paragraph, corresponds to the solubility of the drug within the DES, the total amount of atenolol inserted within the carrier would correspond to 0.86% (*w*/*w*) for the 2% carbomer-based eutectogel and 0.80% (*w*/*w*) for the 10% gelatin-based eutectogel. When we compare this quantity to that reported in the work of Chaerunisaa et al. [47], where atenolol was loaded at 0.5% (*w*/*w*) into the gel, we can assert that the loading capacity of the euctetogel described in our study is higher.

### 3.8. Rheological Study

**Figure 9:**
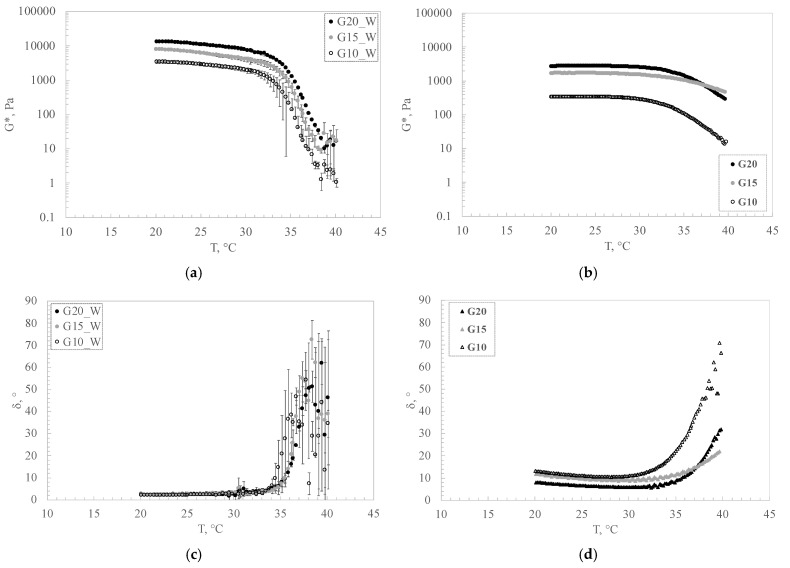
Time cure of gelatin–water-based gels (**a**,**c**) and gelatin–DES-based gels (**b**,**d**) in terms of complex modulus G* (**a**,**b**) and phase angle δ (**c**,**d**).

Samples at three gelatin concentrations were studied using water and DES as solvents. In Figure 9, the behavior of G* and δ as a function of temperature is reported.

From the results obtained, it is possible to observe that the substitution of the same quantity of water with DES causes a decrease in the gel consistency, as shown in Figure 9a,b.

It is also possible to point out that temperature has a different effect on the two types of gels. Specifically speaking, the decrease in the complex modulus and the sharp increment in the phase angle are delayed by using a DES as a solvent. In particular, the water-based gels show a decay of G* of about 3 orders of magnitude compared with the DES samples that show a decrease of about one order of magnitude in the same range of temperature. Then, the destructuration degree for the water-based gels is higher than that for the DES-based ones.

Even if the DES delays the transition with temperature, the delay is not much shifted and the explanation can be found in the nature of gelatin, made from natural biopolymers such as collagen.

In fact, as reported in the literature [48], gelatin sourced from collagen and gels prepared with gelatin lose consistency at body temperature. This thermal property makes gelatin a widely used ingredient in the food industry, and the gelation property, similar to collagen, is important for the structuration of many systems [48].

The gels obtained with carbomer were also studied, both in water and DES.

**Figure 10:**
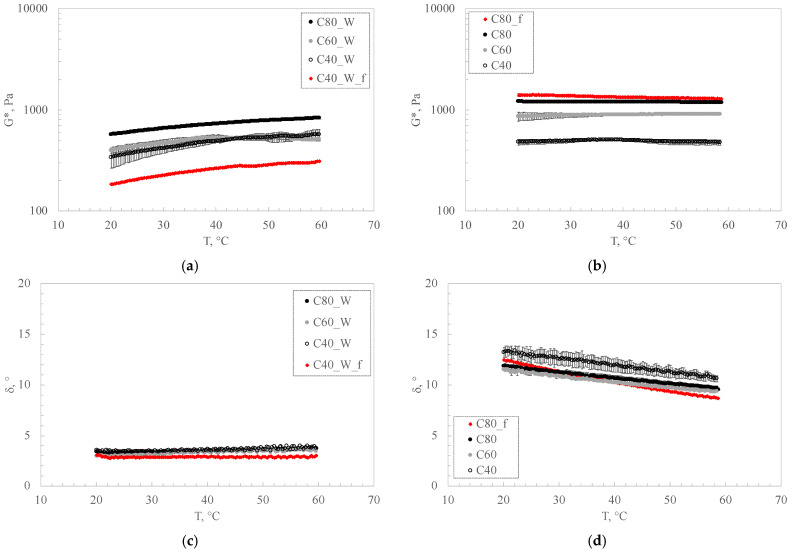
Time cure of carbomer–water-based gels (**a**,**c**) and carbomer–DES-based gels (**b**,**d**) in terms of complex modulus G* (**a**,**b**) and phase angle (**c**,**d**).

As it is possible to observe from Figure 10, the gels are stable in temperature and show a low angle phase at all temperatures. The trends are typical of well-structured and elastic systems, and the behavior can be due to the polymer used to obtain the gels, which is carbomer, a cross-linked polyacrylic acid polymer with high molecular weight and hydrophilic behavior. This polymer forms a physical hydrogel with a three-dimensional polymer network [49]. Also in this case, as for gelatin, water and DES were tested as solvents. For both systems, water- and DES-based, the increase in carbomer leads to more consistent structures. In particular, DES-formed structures tend to have higher G* values than the values obtained for water-based samples, in contrast to the gelatin-based sample trend. The DES-based samples are characterized by high thermal stability, both compared to the water-based samples, which undergo an increase in modulus with temperature, and especially compared to the gelatin samples (Figure 10), which undergo a collapse in modulus and a critical increase in phase angle above a temperature of 35 °C.

The effect of the drug on the weaker carbomer sample (C40_W_F) was tested in a water solvent. The data trend shows a destructuring and weakening effect on the same gel without the drug, probably because of an antagonistic effect that the drug has in forming the network. Drug addition was also tested in the C80 sample (C80_F) in DES. In contrast to the results obtained for the water-based sample (C40_W_F), the C80_F structure underwent a strengthening, probably due to better interaction of the drug with the DES–carbomer network, as is observable from Figure 10.

Rheological analysis suggests that the use of carbomer as a structuring agent is recommended mainly because of its ability to give gels with higher thermal stability. Specifically, the DES-prepared samples showed greater temperature resistance and mechanical stability in the presence of the drug.

Frequency sweep tests were performed on samples at 25 °C and 37 °C, and the results are summarized in Table 1 in terms of A and z parameters. Samples with gelatin and water were fitted by a weak-gel model only at 25 °C because at 37 °C the samples lost the gel structure and the model was not suitable to describe the material.

The increase in gelatin quantity for both solvents results in higher A values and, therefore, in a more consistent sample, while the z parameter increases only for the samples obtained with DES. Specifically speaking, an increment in gelatin is probably not accompanied by correct water content, and this increments only the consistency of the system (A) but not the structure degree because of the insufficient water for the gel formation.

For carbomer samples, it is observed in Table 2 that the increase in concentration results in stronger and more cross-linked structures. Only at 37 °C is there a decrease in the z parameter with concentration, which can be due to the temperature effect, but in any case, the samples show a weak-gel behavior. The DES-based samples result in stronger and lower cross-linking values compared with the water-based carbomer samples.

Finally, the A values obtained for the four formulations studied were compared; it was observed that the trends of A as polymer concentration changes are well described by a straight line whose slope shows how the strength of the system increases with concentration. In particular, the higher the slope of the straight line, the less the amount of polymer needed to create stronger structures.

**Figure 11:**
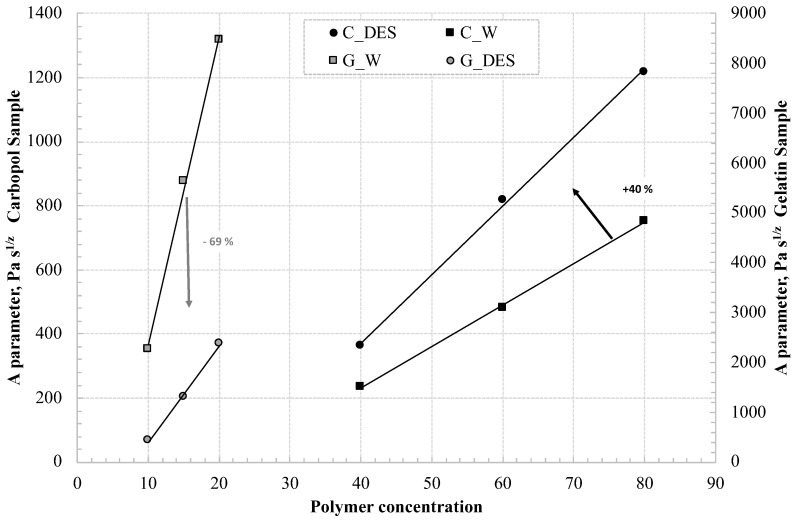
Comparison of A parameter for carbomer and gelatin in water and DES.

A comparison of the straight lines clearly shows that the use of the DES in gelatin-based systems leads to a 69% worsening of the structure while the use of the DES with carbomer yields a 40% improvement in gel strength. This suggests that the use of the DES with carbomer, which is useful in providing thermal stability to the samples, quantitatively improves the structure of the system by 40% (Figure 11). The reported studies were repeated 45 days apart on the same samples, which were properly stored in a refrigerator. Analyses could only be conducted on the eutectogels, as the samples containing water had deteriorated. The results obtained for the eutectogels were superimposable, confirming their stability.

### 3.9. Water Regain Studies

**Figure 12:**
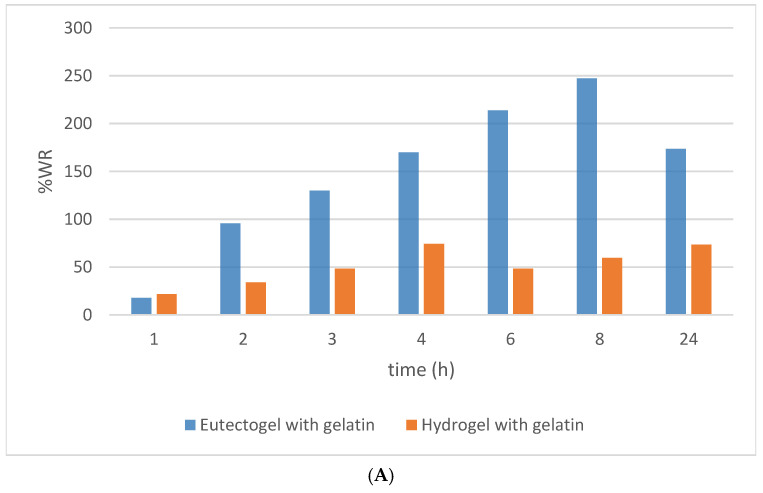

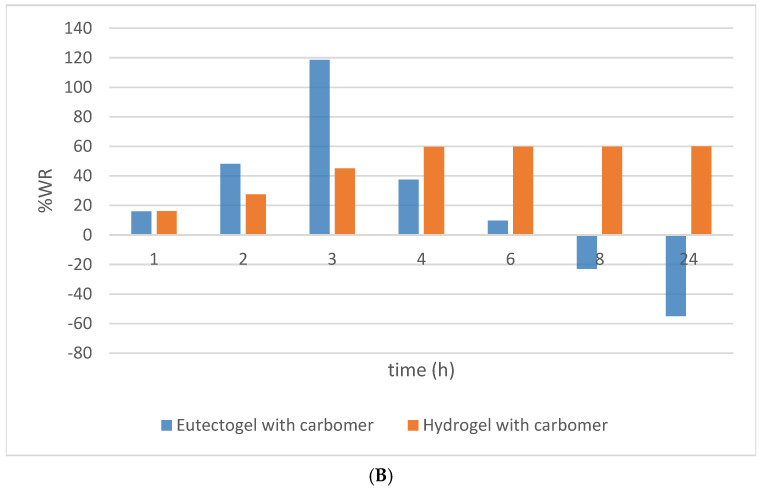
Swelling of eutectogels and hydrogels with gelatin (**A**) and carbomer (**B**).

The results obtained describe a significant difference in the degree of swelling between the eutectogels and the hydrogels (Figure 12). The gelatin-based and carbomer-based hydrogels exhibited similar swelling behavior, with both achieving a swelling ratio between 60 and 70%. However, a differentiated behavior was observed among the eutectogels. The gelatin-based eutectogel showed a swelling capacity up to 250 times greater compared to the carbomer-based eutectogel, which displayed an anti-swelling behavior. This deswelling capability of the eutectogel was observed in the work by Chai et al. [50]. In their study, it was highlighted that deswelling led to a decrease in conductivity and an increase in mechanical properties. This latter aspect was also confirmed in our work by the results of the rheological studies.

### 3.10. In Vitro Skin Permeation

**Figure 13:**
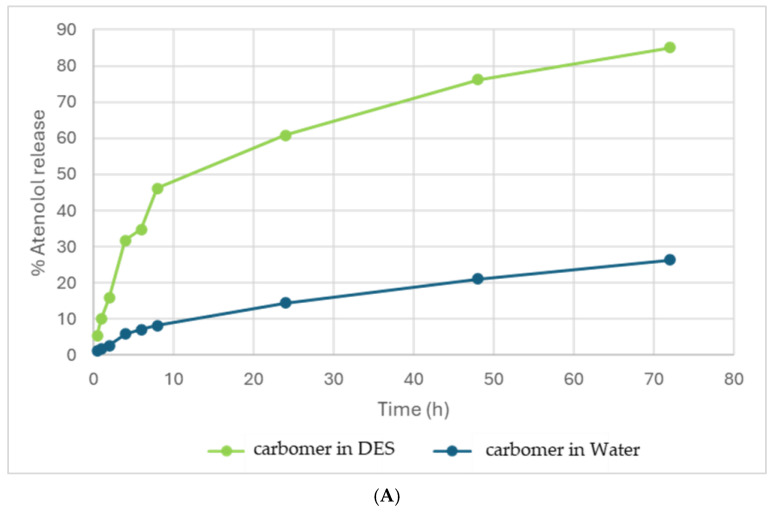

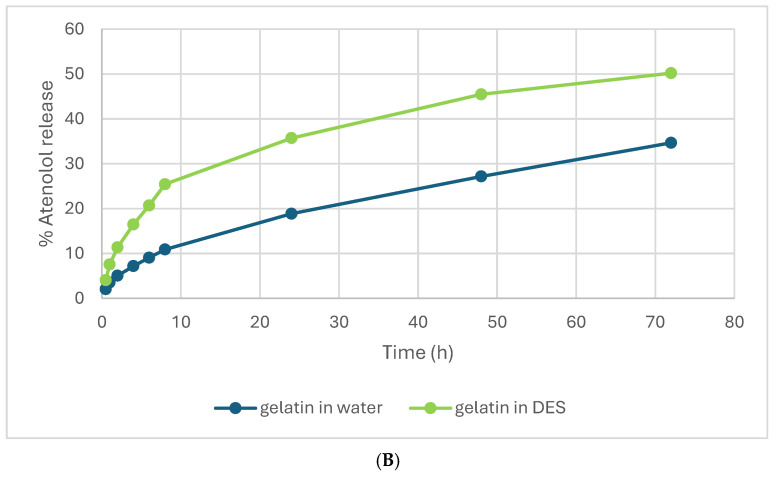
(**A**) Drug permeation by gels based on Carbomer 140–DES and Carbomer 140–water. (**B**) Drug release by gels based on gelatin–DES and gelatin–water.

The in vitro permeation release profile of atenolol from different gels based on the DES and water formed with gelatin and carbomer is reported in Figure 13. It can be observed that gels based on the DES have the capacity to release a large amount of atenolol and achieve controlled release over time. The eutectogel with gelatin released 50.20% after three days while the one with carbomer 85.06% with respect to the gels based on water, of whcih the one with gelatin released 34.67% and the one with carbomer 26.43%. These results demonstrate that eutectogels exhibit better drug delivery and permeation capabilities compared to those based on water, in particular with carbomer.

## 4. Conclusions

In conclusion, the development of eutectogels represents a promising strategy for enhancing the transdermal delivery of atenolol, a beta-blocker. The data confirm the high solubility of atenolol in the deep eutectic solvent (DES) ChCl, providing a solid foundation for its use in drug delivery systems. Eutectogel stability is maintained through extensive hydrogen bonding, as verified through DSC, FTIR, and NMR analyses. The safety and non-toxicity of DES for drug delivery applications were confirmed via MTT assays. The eutectogels, formulated with DES and water using gelling agents like gelatin and carbomer, were characterized, and rheological studies showed that DES–carbomer gels offered enhanced mechanical stability and thermal resistance compared to other formulations. Stability was further supported by reproducible results across trials. Permeation studies demonstrated an 86.5% drug release from DES–carbomer gels, significantly higher than the 26.43% release from water-based gels, indicating that eutectogels improve permeability through their ability to penetrate and transport atenolol across membranes. These findings suggest that DES-based eutectogels offer a superior and viable approach for optimizing atenolol transdermal delivery.

## Figures and Tables

**Table 1:** Weak-gel parameters at 25 °C and 37 °C for gelatin samples with water and DES as solvent.

ID Sample	A, Pa·s^1/z^ 25 °C	z, - 25 °C	A, Pa·s^1/z^ 37 °C	z, - 37 °C
G10	428 ± 20	14.4 ± 0.2	no weak-gel behavior	
G15	1307 ± 40	16 ± 1	267 ± 20	6.6 ± 1
G20	2371 ± 40	23 ± 1	308 ± 10	3.1 ± 0.2
G10_W	2259 ± 20	44 ± 8	no weak-gel behavior	
G15_W	5643 ± 400	20 ± 1	no weak-gel behavior	
G20_W	8464 ± 110	25 ± 1	no weak-gel behavior	

**Table 2:** Weak-gel parameters at 25 °C and 37 °C for carbomer samples with water and DES as solvent.

ID Sample	A, Pa·s^1/z^ 25 °C	z, - 25 °C	A, Pa·s^1/z^ 37 °C	z, - 37 °C
C40	361 ± 4	7.2 ± 0.1	362 ± 2	9 ± 1
C60	818 ± 10	8.6 ± 0.1	893 ± 10	12.2 ± 0.2
C80	1218 ± 14	8.8 ± 0.9	1220 ± 30	10 ± 2
C80_F	1376 ± 4	7.8 ± 0.7	1369 ± 5	8.7 ± 0.2
C40_W	236 ± 15	18 ± 0.2	270 ± 20	56.8 ± 0.1
C60_W	482 ± 40	26 ± 8	565 ±30	30 ± 1
C80_W	752 ± 20	34 ± 3	886 ± 9	19.4 ± 0.4
C40_W_F	303 ± 1	20 ± 3	320 ± 20	59 ± 10

## Data Availability

The original contributions presented in the study are included in the article/Supplementary Material, further inquiries can be directed to the corresponding authors.

## Supplementary Materials

The following supporting information can be downloaded at: https://www.mdpi.com/article/10.3390/pharmaceutics16121552/s1, NMR and FTIR spectra of all compounds.
