# Supplementary material for: Eutectogel-Based Drug Delivery: An Innovative Approach for Atenolol Administration
Source: Pharmaceutics. 2024 Dec 4;16(12):1552. doi: 10.3390/pharmaceutics16121552 (PMC11728620; doi:10.3390/pharmaceutics16121552)

Supplementary Materials

Eutectogel-Based Drug Delivery: An Innovative Approach for Atenolol Administration

Roberta Cassano [1,†], Roberta Sole [1,†], Carlo Siciliano [1], Noemi Baldino [2], Olga Mileti [2], Debora Procopio [1], Federica Curcio [1], Gabriella Calviello [3,4], Simona Serini [3,4], Sonia Trombino [1,*] and Maria Luisa Di Gioia [1,*]

NMR experimental details

All spectra were recorded on a Bruker Advance 300 Ultrashielded NMR spectrometer, at 300.102 MHz for ^{1}H and 75.025 MHz for ^{13}C. Native pulse sequences and elaboration programs from Bruker were applied to collect all spectral data. All analyses were performed using DMSO-d_6 as the deuterated solvent (purchased from Sigma-Aldrich, isotopic purity 99.9 %), without TMS addition. Spectra were calibrated on the resonances of the residual solvent (2.51 ppm, central line of the quintet for ^{1}H spectra; 40.0 ppm, central line of the septet for ^{13}C spectra). Temperature of the probe was fixed at 303 K for all spectra. Each sample was prepared by dissolving aliquots of samples (30 mg) in 0.75 mL of the deuterated solvent. Chemical shifts (δ) are reported in ppm and referred to the calibration frequencies of the deuterated solvents, and coupling constants (J) are reported in Hz.

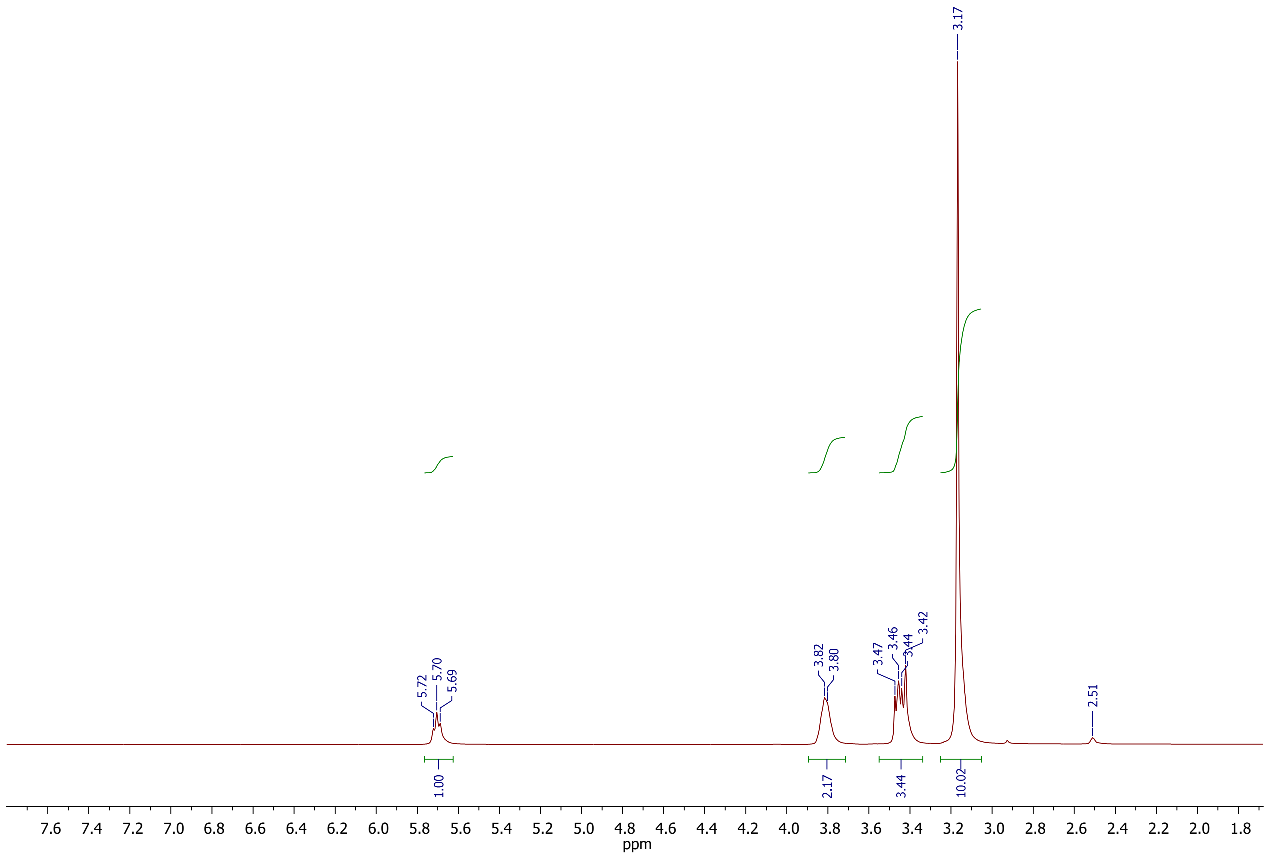

^{1}H NMR spectrum of a standard sample of choline chloride.

^{1}H NMR (DMSO-d$_6$), δ (ppm): 5.70 (t, J = 6 Hz, 1 H; OH), 3.75-3.83 (m, 2 H; OCH_2), 3.35-3.45 (m, 2 H; NCH_2), 3.17 (s, 9 H; CH_3).

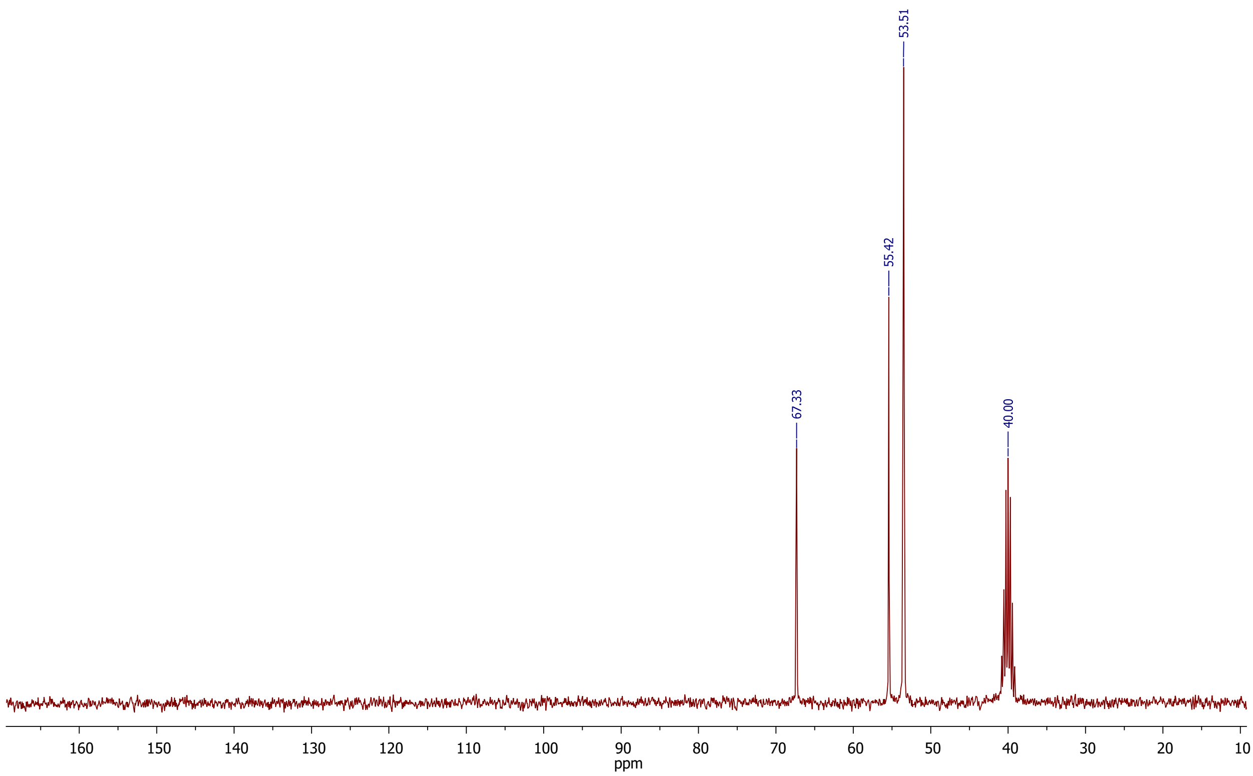

^{13}C NMR spectrum of a standard sample of choline chloride.

^{13}C NMR (DMSO-d_6), δ (ppm): 67.3 (O*C*H_2), 55.4 (N*C*H_2), 53.5 (CH_3).

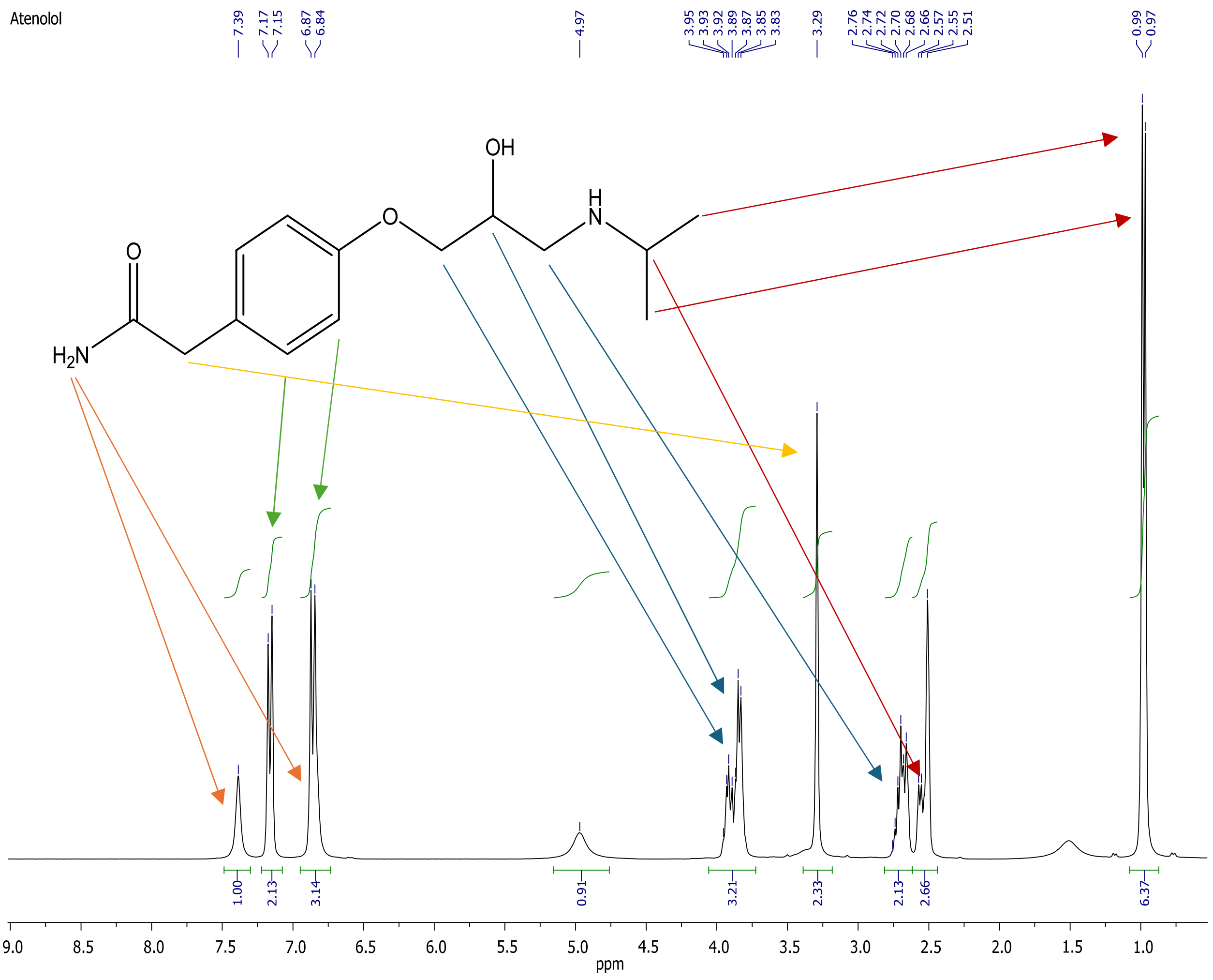

^{1}H NMR spectrum of atenolol.

^{1}H NMR (DMSO-d$_6$), *δ* (ppm): 7.39 (bs, 1 H; NH_2), 7.16 (d, *J* = 6 Hz, 2 H; ArH), 6.86 (d, *J* = 6 Hz, 3 H; ArH and NH_2), 4.97 (bs, 1H, OH), 3.76-3.89 (2 m, 3 H; C*H*OH and OCH_2), 3.29 (s, 2 H, CH_2CO), 2.63-2.76 (m, 2 H; C*H*$_2$NH and NHC*H*), 2.49-2.61 (m, 1 H; C*H*$_2$NH), 1.50 (bs, 1 H; NH), 0.98 (d, *J* = 6 Hz, 9 H; CH_3).

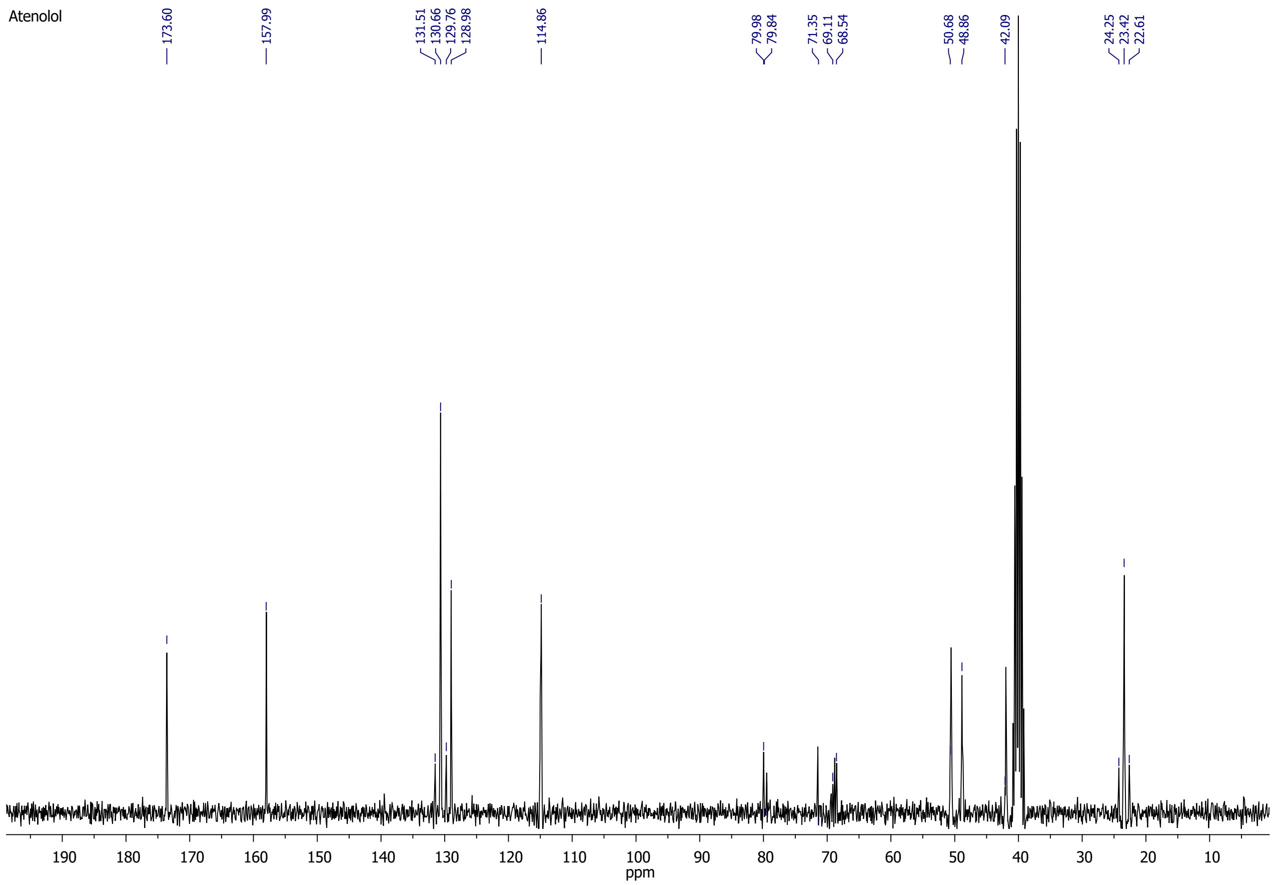

^{13}C NMR spectrum of atenolol.

^{13}C NMR (DMSO-d$_6$), δ (ppm): 173.6 (CO), 157.9 (CAr), 130.6 (CHAr), 128.9 (CAr), 114.8 (CHAr), 79.9 (*C*H$_2$O), 71.3 (*C*HOH), 50.7 [*C*H(CH$_3$)], 48.8 (*C*H$_2$NH), 42.1 (*C*H$_2$CO), 23.4 (CH$_3$).

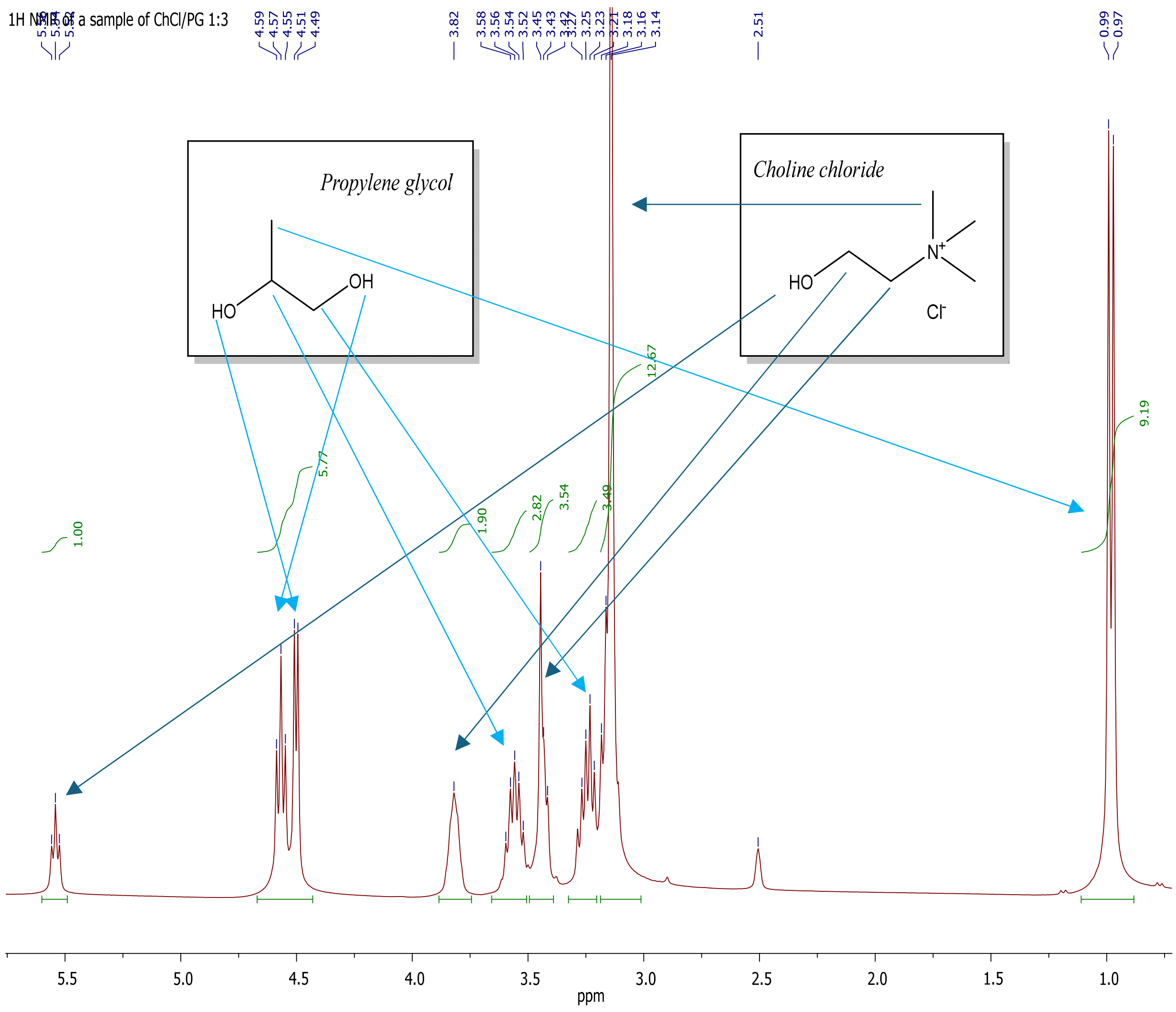

^{1}H NMR spectrum of ChCl:PG DES

(ChCl:PG) DES. ^{1}H NMR (δ): 5.55 (t, *J* = 3 Hz, 1H, OH ChCl), 4.57 (t, *J* = 6 Hz, 1H, CH_2OH PG), 4.51 (d, *J* = 3 Hz, 1H, CHOH PG), 3.75-3.87 (m, 2H, CH_2OH ChCl), 3.48-3.62 (m, 1H, CH PG), 3.40-3.47 (m, 2H, CH_2N^+ PG), 3.20-3.32 (m, 1H, CH_2 PG), 3.15 (s, 9H, CH_3 ChCl), 3.10-3.20 (m, 1H, CH_2 PG), 0.99 (d, *J* = 6 H, 3H, CH_3 PG) ppm;

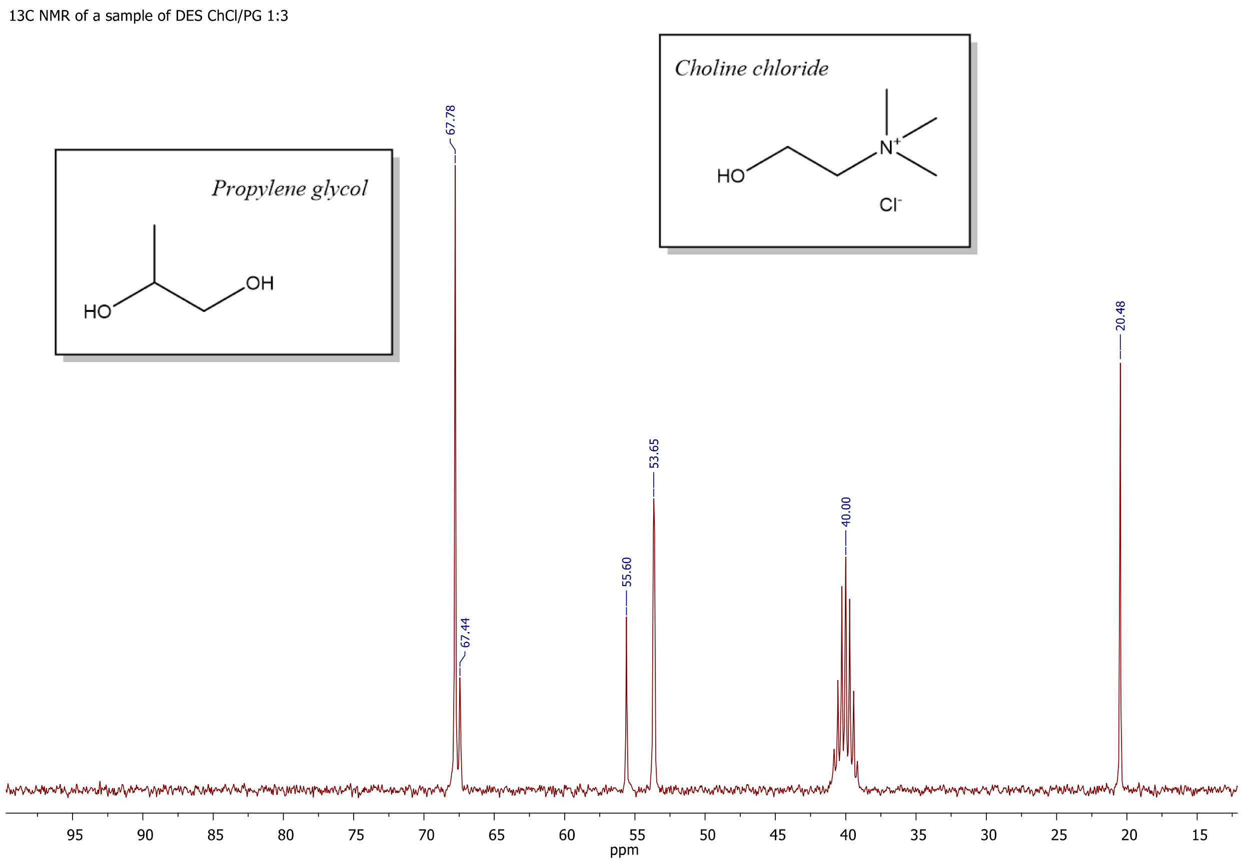

^{13}C NMR spectrum of ChCl:PG DES

^{13}C NMR (δ): 67.78, 67.44, 55.60, 53.64, 20.48.

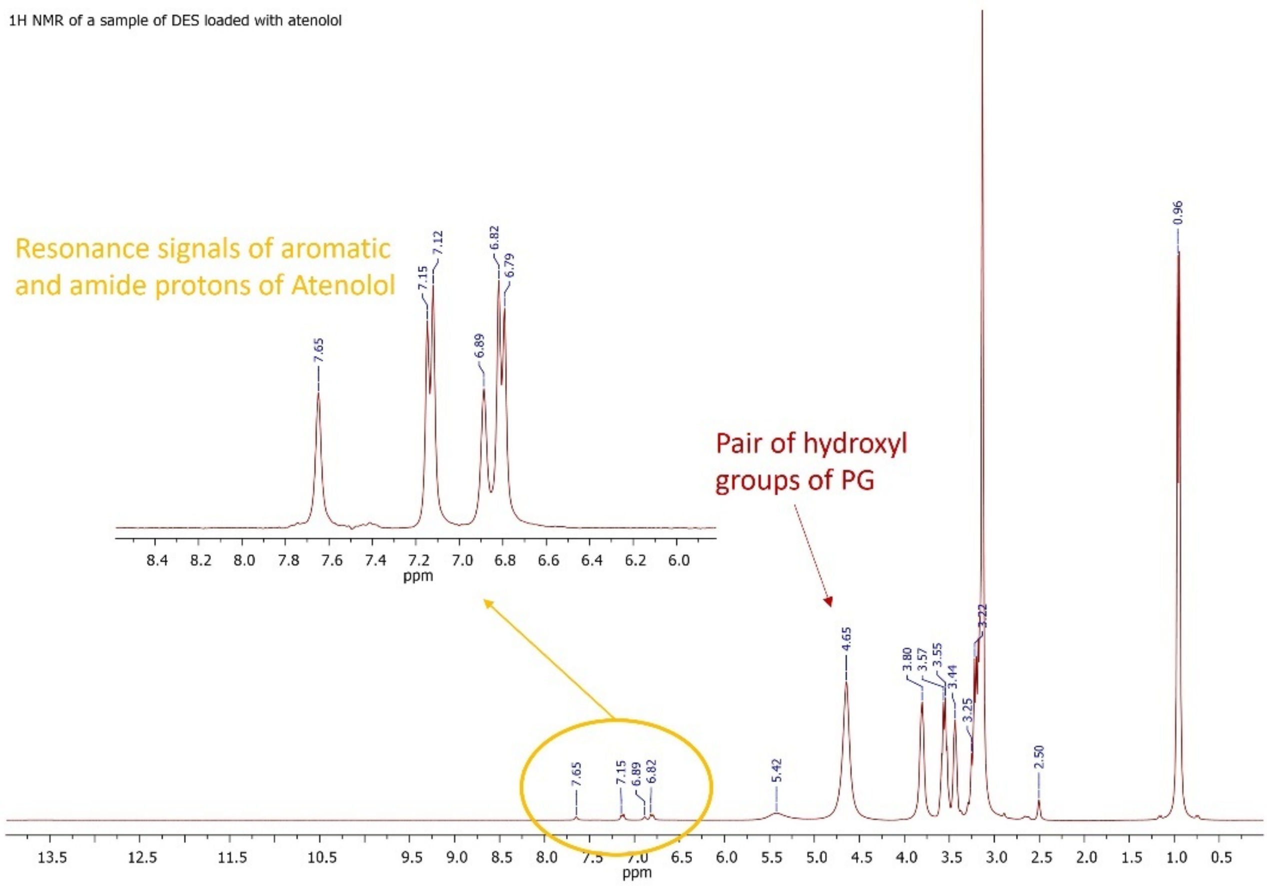

^{1}H NMR of atenolol loaded ChCl:PG DES

7.65 (br s, 1H, NH_2CO ATL), 7.13 (d, 2H, J= 9Hz, ArH ATL),6.89 (br s, 1H, NH_2CO ATL), 6.81 (d, 2H, J= 9Hz, ArH ATL), 5.42 (br s, OH ChCl), 4.65, (br s, 2H, OH PG, OH PG), 3.80 (m, 4H, CH2 ChCl and CH_2OH PG), 3.57-3.55 (m, 4H, CHOH and OCH_2 ATL, CHOH PG), 2.52-2.49 (m, 3H, CH_2NH, NHCH), 3.44 (m, 2H, CH_2 ChCl), 3.23 (m, 2H, CH_2CO, (s, 3.31 (s, 9H, $N(CH_3)_3$) , 0.96 (d, J = 7.20 Hz , $CH(CH_3)_2$).

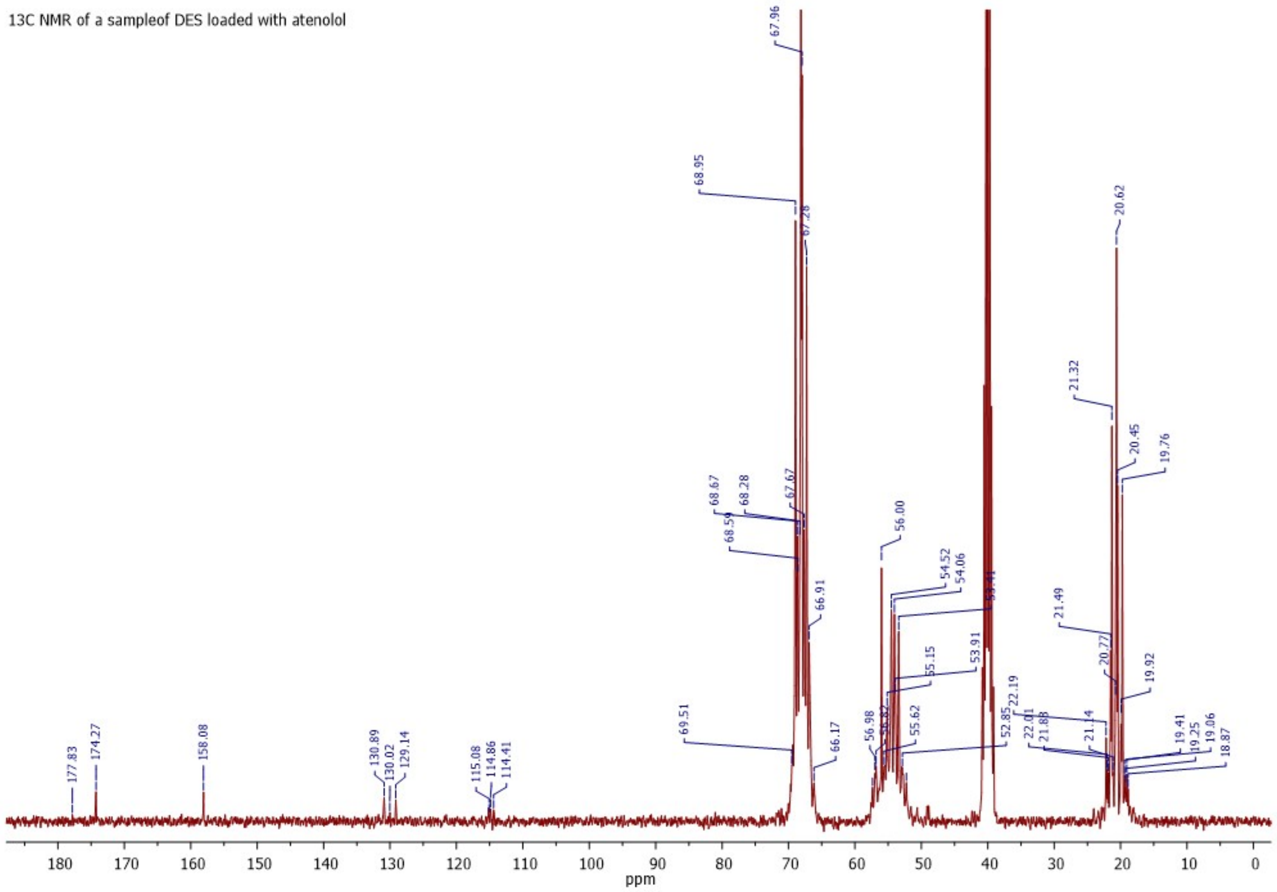

13C NMR of atenolol loaded ChCl:PG DES

^{13}C NMR (δ): 174.27, 158.08, 130.89, 115.08, 69.51, 68.95, 68.67, 68.59, 68.95. 68.28, 67.96, 56.00, 54.06, 52.85, 20.62, 19.76.

FT -IR

Propylene Glycol

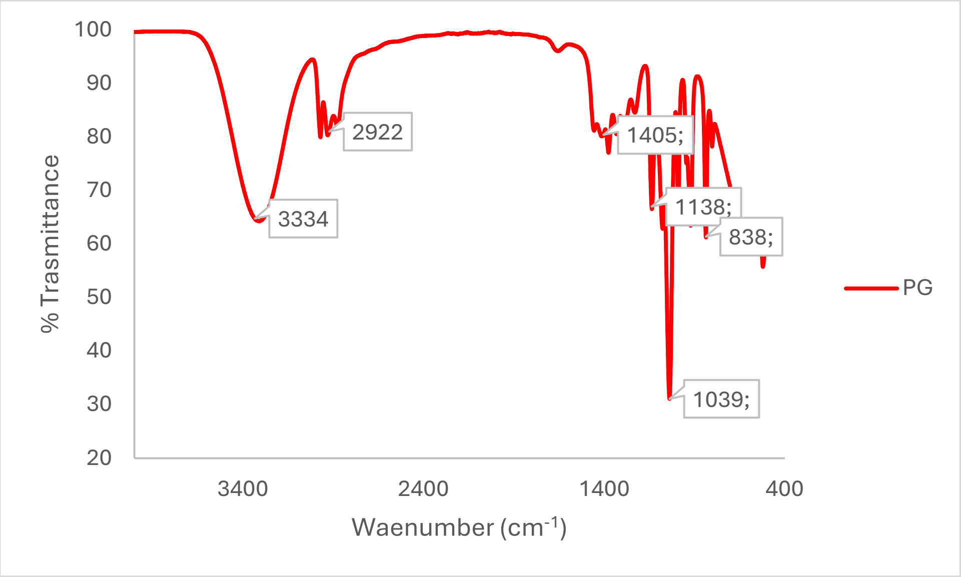

Choline Chloride

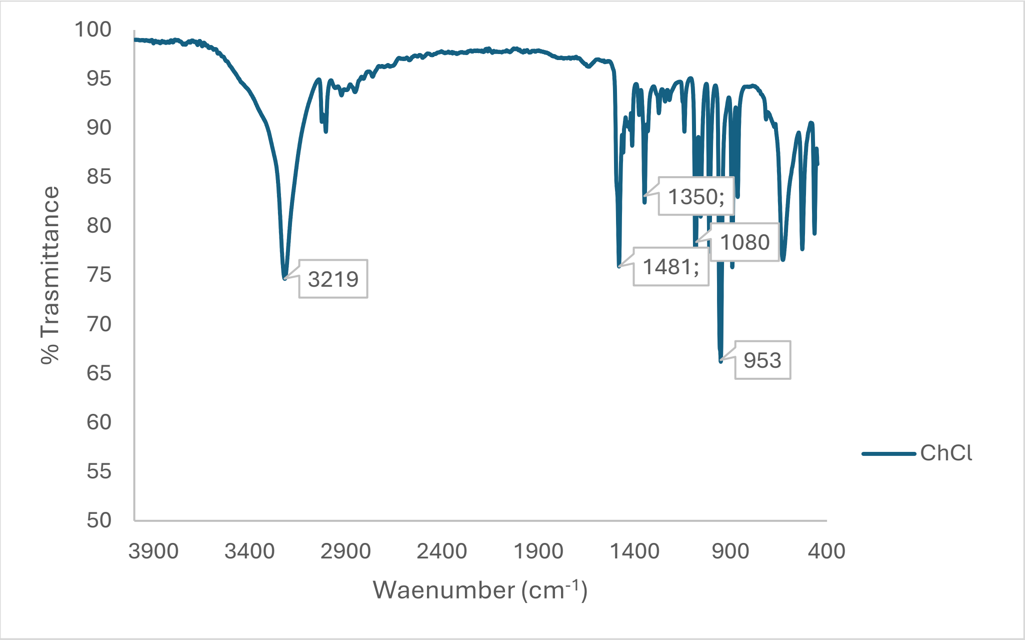

ChCl:PG DES

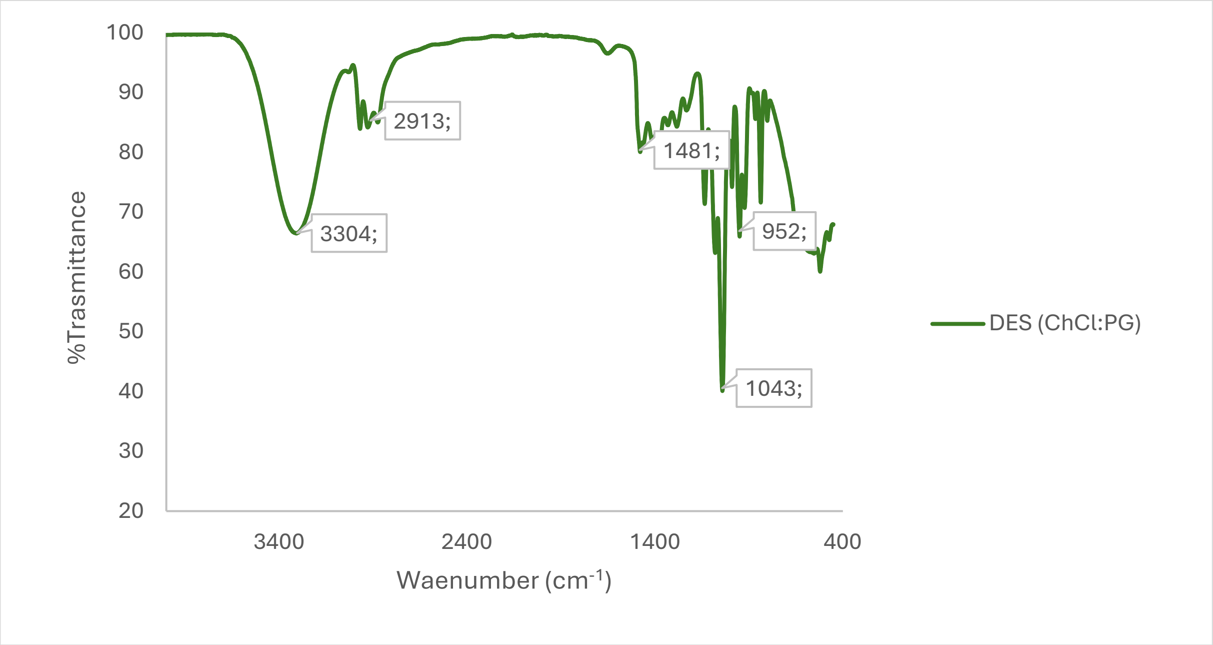

Atenolol

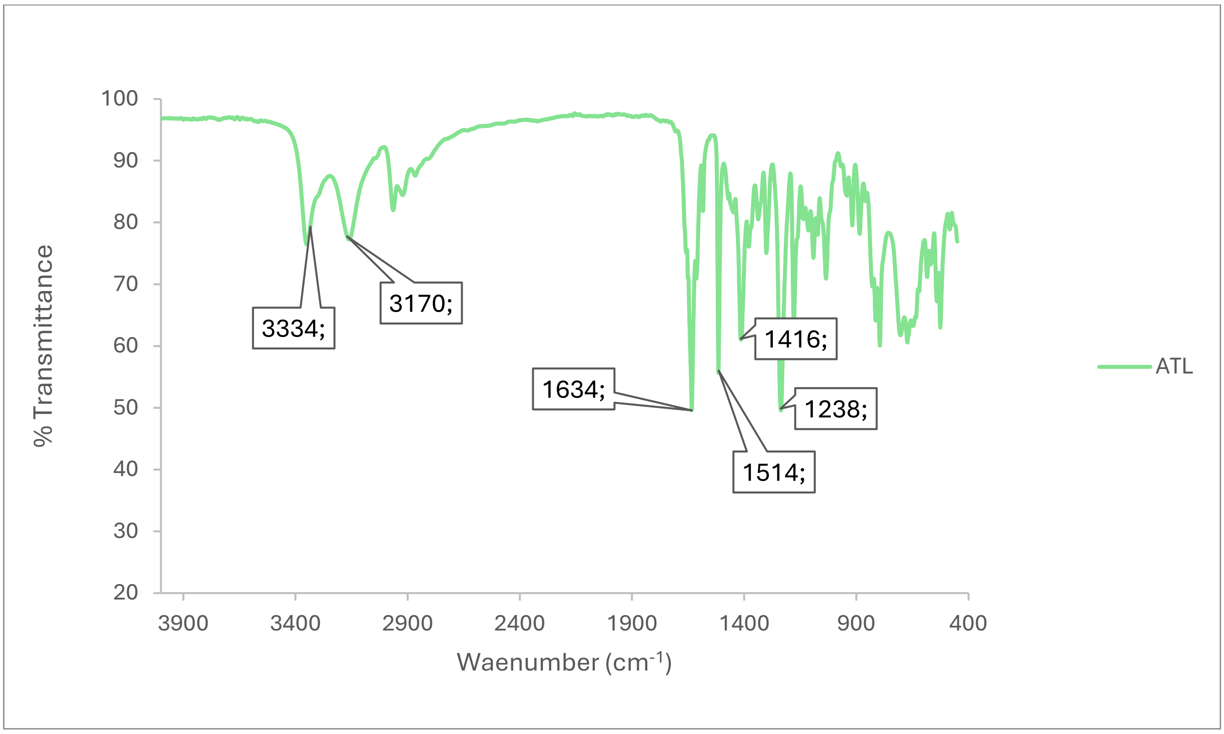

Atenolol loaded DES

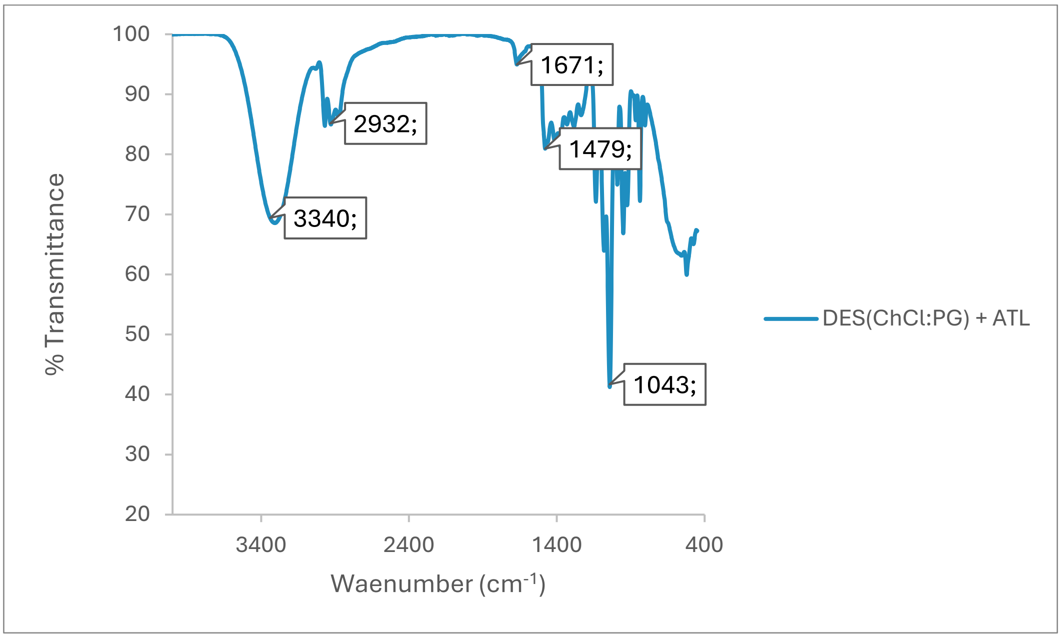

Supplement: Supplementary file 1 [file pharmaceutics-16-01552-s001.zip › pharmaceutics-3250685-supplementary.pdf]
